# Trabecular bone score and phalangeal quantitative ultrasound are associated with muscle strength and fracture risk in hemodialysis patients

**DOI:** 10.3389/fendo.2022.940040

**Published:** 2022-09-07

**Authors:** Antonino Catalano, Agostino Gaudio, Federica Bellone, Mattia Miriam La Fauci, Anastasia Xourafa, Guido Gembillo, Giorgio Basile, Giuseppe Natale, Giovanni Squadrito, Francesco Corica, Nunziata Morabito, Domenico Santoro

**Affiliations:** ^1^ Department of Clinical and Experimental Medicine, University Hospital of Messina, Messina, Italy; ^2^ Department of Clinical and Experimental Medicine, University Hospital of Catania, Catania, Italy; ^3^ Mineral Metabolism and Nephrology Clinic of Vibo Valentia Hospital, Vibo Valentia, Italy

**Keywords:** fracture, osteoporosis, hemodialysis, bone mineral density (BMD), trabecular bone score (TBS), chronic kidney disease-mineral bone disorder (CKD-MBD), quantitative ultrasound (QUS), handgrip strength

## Abstract

There is growing interest in the relationship between chronic kidney disease (CKD) and fragility fracture risk. Bone mineral density (BMD) is a major determinant of bone strength, although its role as a predictor of fracture in advanced CKD and hemodialysis is still under debate. We aimed to further investigate surrogates of bone quality and their associations with muscle strength and fracture risk in hemodialysis. Multiple clinical risk factors for fracture and an estimated 10-year probability of fracture, BMD at lumbar spine and femur, trabecular bone score (TBS), X-ray vertebral morphometry, phalangeal bone quantitative ultrasonography (QUS), tibial pulse-echo ultrasonography (PEUS), and handgrip strength were evaluated in a setting of hemodialysis patients in treatment with acetate-free biofiltration (AFB) or bicarbonate hemodialysis. The bone ultrasound measurements, both at phalangeal and tibial sites, were significantly associated with lumbar and femoral DXA values. Handgrip strength was significantly associated with the 10-year probability of fracture (*r* = −0.57, *p* < 0.001 for major fractures and *r* = −0.53, *p* < 0.001 for hip fracture, respectively), with femur neck, total femur, and L1–L4 BMD values (*r* = 0.47, *p* = 0.04; *r* = 0.48, *p* = 0.02; *r* = 0.58, *p* = 0.007, respectively), with TBS at the lumbar spine (*r* = 0.71, *p* < 0.001) and with the phalangeal QUS measure of AD-SoS (*r* = 0.369, *p* = 0.023). In the hemodialysis group, 10 participants (24.3%) reported at least one morphometric vertebral fracture (Vfx); conversely, only six participants (15%) showed Vfx in the control group. In the hemodialysis group, participants with Vfx compared with participants without Vfx reported significantly different TBS, bone transmission time (BTT), cortical thickness, and handgrip strength (*p* < 0.05). At multiple regression analysis, by identifying as dependent variable the 10-year fracture risk for major fracture, after correcting for age, BMI, time since dialysis, AD-SoS, cortical bone thickness, and handgrip strength, only BTT (*β* = −15.21, SE = 5.91, *p* = 0.02) and TBS (*β* = −54.69, SE = 21.88, *p* = 0.02) turned out as independently associated with fracture risk. In conclusion, hemodialysis patients showed a higher fracture risk and lower surrogate indices of bone strength as TBS and QUS parameters. In this cohort of patients, handgrip strength measurements appeared to be a useful instrument to identify high-fracture-risk subjects.

## Introduction

Fragility fractures are common in patients with chronic kidney disease (CKD), particularly in the end-stage, and are often associated with excess morbidity and mortality (1).

Disorders of mineral metabolism and bone microarchitecture arise early in the CKD course and gradually worsen along with the deterioration of kidney function; as a result, half of the CKD patients initiating dialysis treatment have already had a fracture (2). Particularly, the risk of fracture at the femur site is approximately fourfold greater in patients with end-stage disease than in the general population fracture rate (1, 3). Incident femur fractures increase the mortality rate by two times in end-stage CKD patients compared with matched end-stage CKD patients without fractures (3). Vertebral fractures (Vfx) are also a common occurrence in CKD. In fact, VFx was revealed in approximately one out of five patients with 3–5 stage CKD; the prevalence of Vfs was correlated with poor survival and raised as an independent predictor of all-cause mortality (4).

Patients with CKD who fracture because of low-grade trauma, in accordance with the Kidney Disease Improving Global Outcomes (KDIGO) classification, present a CKD-Mineral and Bone Disorder (CKD-MBD); this latter is a systemic disorder of mineral and bone metabolism manifested by either one or a combination of the following: vascular or other soft tissue calcification; abnormalities of calcium, phosphorus, parathyroid hormone (PTH), or vitamin D metabolism; or abnormalities in bone turnover, mineralization, volume, linear growth, or strength (5).

Low bone mineral density (BMD), as measured by dual-energy X-ray absorptiometry (DXA), is a recognized major predictor of fracture in the general population. However, studies have reported conflicting results on the association between DXA and fracture in end-stage CKD (2). Moreover, by considering a large population (*n* = 13,848) from the Third National Health and Nutrition Examination Survey (NHANES III; 1988–1994), it has been observed that while BMD decreased along with decreasing kidney function (based on estimated creatinine clearance (eCcr) calculated using the Cockcroft–Gault (CG) formula), after controlling for only sex, age, and weight, any negative association between BMD and kidney function was extinguished (6). Because of the possibility of falsely elevated BMD values due to vascular or paraspinal tissue calcifications or age-related changes in the lumbar spine, the prevalence of osteoporosis in patients with advanced CKD may be undervalued based on a T-score value of ≤ −2.5 standard deviations (SD) if BMD is measured at the lumbar spine (6–9). These findings demonstrate that BMD testing to estimate fracture risk might not always be advantageous in advanced stages of CKD. However, the 2017 KDIGO guidelines recommend that in patients with evidence of CKD-MBD and/or risk factors for osteoporosis, BMD measurement of bone density is expected to impact treatment decisions (10). The Fracture Risk Assessment Tool (FRAX) index is a validated resource for the 10-year prediction of major osteoporotic fractures, assessing clinical risk factors together with BMD, particularly in CKD stages 2–5 (11, 12). FRAX was able to discriminate fracture status among men and women with CKD but performed no better than femoral BMD alone (12). In particular, in a cohort of 718 hemodialysis patients who were followed up for 2 years, Przedlacki et al. identified the FRAX value of 5% as the prognostic threshold for an increased risk of major osteoporotic fracture (13).

Besides the bone density impairment, CKD patients are more likely to fracture because of the deterioration of bone quality. Bone strength, in fact, also depends on bone microarchitecture and materials’ properties that cannot be measured by DXA (5, 14).

The trabecular bone score (TBS) represents a novel texture parameter reflecting bone microarchitecture. It results from the computed analysis of gray-level changes in pixels of lumbar spine DXA images and may contribute to fracture risk assessment (15–17). Moreover, bone quantitative ultrasonography (QUS) has been proposed to explore fracture risk, taking into account that QUS measurements are related to some physical properties of bone tissue (e.g., structure and elasticity) that contribute to bone strength. Previous studies correlated QUS with DXA measurements, prevalent Vfx, and risk of future fractures (11, 14, 18–20). Additionally, studies using pulse-echo ultrasonography (PEUS), a novel ultrasound method that measures the thickness of cortical bone, have shown a significant correlation between density index (DI), a PEUS-derived parameter, and BMD at the hip in postmenopausal women (21, 22).

High fracture risk in hemodialysis patients is also likely to be related to a propensity to falls due to poor muscle strength and impaired balance secondary to poor nutrition, inactivity, myopathy, and peripheral neuropathy (23). Previous studies have studied the positive effects of muscle function on bone health, and the relationship between muscle strength and BMD has been highlighted (24–26). However, very little evidence exists on this association in dialysis patients (27, 28), and no data are available on the association between muscle strength and TBS or QUS/PEUS measurements intended as surrogates of bone quality or bone strength.

The primary aim of this study was to investigate bone health in hemodialysis patients with both DXA and bone ultrasonometry tools, and the secondary purpose was to measure muscle strength and investigate the possible associations between muscle and surrogates of bone strength.

## Materials and methods

### Participants

This cross-sectional study included adult (>18 years) Caucasian subjects with CKD 5D, referred to the Nephrology Unit of the Department of Medical Sciences, University Hospital of Messina (Messina, Italy). The enrolled patients were selected from the entire hemodialysis cohort followed up at our University Center. The research protocol was approved by the Local Ethics Committee for Medical Research, Messina University Hospital “G. Martino” and carried out in accordance with the 1964 Declaration of Helsinki and its later amendments.

Exclusion criteria were dementia or cognitive impairment; history of cancer; hypo- or hyperthyroidism; malabsorption diseases; and previous use of active bone agents, including denosumab, selective estrogen receptor modulators, and strontium ranelate. Controls were consecutively enrolled from outpatients referred to the metabolic bone disease service of the Geriatric Unit of the Department of Medical Sciences, University Hospital of Messina (Messina, Italy).

All the participants signed an informed consent before entering the study.

### Clinical evaluation

Height and weight were measured at baseline according to standard procedures, and BMI was calculated as weight in kilograms divided by the square of height in meters (kg/m²).

Fracture risk assessment was estimated by the Fracture Risk Assessment Tool (FRAX^®^), which is a computer-based algorithm (http://www.shef.ac.uk/FRAX) that calculates the 10-year probability of a major fracture (hip, clinical spine, humerus, or wrist fracture) and the 10-year probability of hip fracture. According to FRAX, calibrated for Italian subjects, fracture risk was derived from age, BMI, and dichotomized risk factors comprising prior fragility fracture, parental history of hip fracture, current tobacco smoking, and exposure to oral glucocorticoids, rheumatoid arthritis, other causes of secondary osteoporosis, and alcohol consumption. FRAX score was calculated without considering BMD (11).

A trainer examiner assessed muscle strength by measuring handgrip strength using a Jamar dynamometer, following a standardized protocol consisting of three consecutive grip strength measurements with the second handle position of the device for each hand, with a rest period of 30 s between successive attempts. Maximal handgrip strength was recorded.

### Bone status evaluation

BMD was assessed by a DXA densitometer (Hologic Discovery Wi) at the lumbar spine (L1–L4) and femoral sites (neck and total femur). The DXA densitometer was calibrated daily according to the manufacturer’s instructions, and its coefficient of variation (CV) was 0.5% at the lumbar spine and femoral site. In addition, we investigated TBS through further evaluation of DXA images by iNsight software (version 3.0; Medimaps Group, Geneva, Switzerland). TBS was evaluated considering the variogram of the trabecular bone–projected image, calculated as the sum of the squared gray-level differences between pixels at a specific distance and angle. TBS was then calculated as the slope of the log–log transform of this variogram (29).

To assess bone status, we also performed QUS measurements at the proximal phalangeal metaphysis of the last four fingers of the nondominant hand using a DBM Sonic Bone Profiler (Igea, Carpi, Italy) as previously described (30). Briefly, amplitude-dependent speed of sound (AD-SoS), bone transmission time (BTT), fast-wave amplitude (FWA), signal dynamic (SDy), and the derived ultrasound bone profile index (UBPI) (UBPI = −(0.0018 × SDy − 0.0560 × FWA 0.0560 − 1.1467 × BTT + 3.0300)) were the considered QUS variables.

Moreover, ultrasound measurements were conducted at the tibial site by using a PEUS device (Bindex^®^; model BI-100, Bone Index Finland Ltd., Kuopio, Finland, Software v.2.0), consisting of a pulser unit plugged into the USB port of a laptop and a focused ultrasound probe (3.0 MHz nominal center frequency). The cortical thickness (CTh) was estimated at one-third of the proximal tibia, meaning a third of the distance from the knee joint space to the medial malleolus, by multiplying the time of flight between the ultrasound echoes from the periosteal and endosteal surfaces by the speed of sound (SOS). An appropriate ruler was used to indicate the measurement at exactly one-third of the tibia length. From CTh, age, weight, and height, a DI (g/cm^2^) was automatically calculated (27).

### Vertebral fracture assessment

All the participants underwent a lateral thoracic and lumbar spine X-ray scan to evaluate morphometric Vfx. As previously described, Vfx was diagnosed if a vertebral body had at least a 20% height reduction in the anterior, middle, or posterior height compared with the same or adjacent vertebra (31).

### Hemodialysis treatment characteristics

Patients on hemodialysis were regularly treated with the Artis Physio system (Baxter Healthcare Corporation, One Baxter Parkway, Deerfield, IL 60015 USA). The scheduled hemodialysis sessions were performed three times a week using a polyethersulfone membrane. The dialysis bath (Safebag KV 95G, Hospal Spa, Bologna, Italy) had the following composition: NaCl (284.3 g/L), KCl (19.57 g/L), CaCl_2_ (7.72 g/L), MgCl_2_ (2.63 g/L), and glucose (35 g/L). Hemodialysis participants were both men and women who had been treated with hemodialysis for 4.5 ± 4 years. All patients had achieved stable dry weight for at least 3 months and had an adequate dialysis delivery [Kt/V 1.2 (1.1 to 1.2)].

### Statistical analysis

Statistical analyses were performed using MedCalc software (version 20.113). Data were reported as means ± SD or median (IQR) for continuous variables and percentages for categorical variables. The Kolmogorov–Smirnov test confirmed the normal distribution of values. Student’s *t*-tests for unpaired observations and the Mann–Whitney test were used as appropriate. Fisher’s exact test was used to calculate differences in categorical variables. Spearman’s coefficient verified the degree of association between two variables. Multiple regression analysis was performed to analyze the relationship between a dependent variable and one or more independent variables. All reported *p*-values were two-sided, and values of *p* < 0.05 were considered to indicate statistical significance.

## Results

The main clinical features of participants are shown in **Table 1**. Hemodialysis patients and controls did not differ for age and BMI but significantly differed for FRAX-derived 10-year hip fracture risk, BMD values at the lumbar spine, TBS, QUS parameters, PTH, phosphorus, and hemoglobin levels, as shown in **Table 1**. Handgrip strength was also significantly different between hemodialysis patients and controls (**Table 1**).

**Table 1:** Main clinical features of hemodialysis patients and controls.

	Patients (n = 41)	Controls (n = 44)	p-value
Age (years)	74.23 ± 11.7	71.82 ± 8.1	0.28
BMI (kg/m^2^)	26.21 ± 6.19	26.45 ± 4.16	0.83
Males (%)	56	57	
Time since hemodialysis (years)	4.5 ± 4	–	
Acetate-free biofiltration (%)	83	–	
Bicarbonate dialysis (%)	17	–	
Kt/V	1.2 (1.1 to 1.2)	–	
Calcium (g/dl)	8.9 ± 1	9.1 ± 1.2	0.40
Phosphorus (g/dl)	4.8 ± 1.3	3.2 ± 0.5	<0.001
Parathyroid hormone (pg/ml)	224.2 ± 163.9	45.2 ± 12.3	<0.001
Hemoglobin (g/dl)	11 ± 4.5	13.5 ± 1.3	<0.001
Albumin (g/dl)	3.8 ± 0.4	3.7 ± 0.3	0.19
**Dual-energy X-ray absorptiometry**			
L1-L4 BMD (g/cm^2^)	0.9 ± 0.14	0.79 ± 0.18	0.002
L1-L4 T-score (SD)	−1.45 ± 1.12	−2.51 ± 0.32	<0.001
Femur neck BMD (g/cm^2^)	0.58 ± 0.06	0.59 ± 0.08	0.52
Femur neck T-score (SD)	−2.5 ± 0.5	−2.4 ± 0.13	0.22
Total femur BMD (g/cm^2^)	0.74 ± 0.09	0.73 ± 0.1	0.6
Total femur T-score (SD)	−1.78 ± 0.62	−1.98 ± 0.22	0.06
Trabecular bone score	1.21 ± 0.12	1.30 ± 0.07	0.0001
**Phalangeal QUS**			
AD-SoS (m/s)	1,636.3 ± 63.6	1,874.42 ± 107.84	<0.0001
UBPI (U)	0.15 ± 0.08	0.32 ± 0.18	<0.0001
BTT (m/s)	1.05 ± 0.41	1.19 ± 0.15	0.04
**Tibial PEUS**			
Cortical thickness (mm)	2.8 ± 1.2	2.7 ± 1	0.6
Density index (g/cm^2^)	0.79 ± 0.1	0.81 ± 0.1	0.37
**Muscle strength**			
Handgrip strength (kg)	18 ± 9.6	22.5 ± 8.2	0.02
**10-year probability of fracture**			
Major fracture (%)	12.7 ± 9.4	10.2 ± 4.4	0.13
Hip fracture (%)	6.6 ± 7.13	3.4 ± 2	0.001

The associations between DXA, QUS, and PEUS variables in the hemodialysis group are shown in **Table 2**. Ultrasound measurements, both at phalangeal (i.e., AD-SoS, UBPI, BTT) and tibial sites (i.e., Cth, DI), were all significantly associated with lumbar and femoral BMD values (*p* < 0.05). Moreover, phalangeal AD-SoS was significantly related to tibial cortical thickness and DI (*r* = 0.36, *p* = 0.02 and *r* = 0.49, *p* = 0.004, respectively). The same association was observed between phalangeal BTT with Cth and DI at the tibial site (*r* = 0.41, *p* = 0.04 and *r* = 0.4, *p* = 0.04, respectively).

**Table 2:** Coefficients of association (*r*) between DXA and ultrasound measurements in hemodialysis patients.

DXA measurements	Ultrasound measurements				
	AD-SoS	UBPI	BTT	DI	Cth
Femur neck BMD	0.46^*^	0.49	0.81^*^	0.508^*^	0.387
Total femur BMD	0.88^*^	0.62^*^	0.82^*^	0.376	0.201
Lumbar spine BMD	0.72^*^	0.67^*^	0.61^*^	0.644^*^	0.534^*^
Trabecular bone score	0.42^*^	0.62^*^	0.07	0.07	0.08

^*^ p < 0.05, statistical significance. BMD, bone mineral density; AD-SoS, amplitude-dependent speed of sound; BTT, bone transmission time; UBPI, ultrasound bone profile index; DI, density index; Cth, cortical thickness.

The 10-year probability of major fracture and hip fracture was significantly associated with femur neck BMD (*r* = −0.59, *p* = 0.003 and *r* = −0.581, *p* = 0.005, respectively), DXA-derived TBS at the lumbar spine (*r* = −0.45, *p* = 0.03), and QUS measurements; in particular, AD-SoS and BTT were associated with the 10-year probability of major osteoporotic fracture (*r* = −0.37, *p* = 0.01 and *r* = −0.43, *p* = 0.03), whereas DI was associated with the 10-year probability of hip fracture (*r* = −0.36, *p* = 0.02). Handgrip strength measurements were significantly associated with the 10-year probability of fracture (*r* = −0.57, *p* < 0.001 for major fractures and *r* = −0.53, *p* < 0.001 for hip fracture, respectively), with femur neck, total femur, and L1–L4 BMD values (*r* = 0.47, *p* = 0.04; *r* = 0.48, *p* = 0.02; *r* = 0.58, *p* = 0.007, respectively), with DXA-derived TBS at the lumbar spine (*r* = 0.71, *p* < 0.001) and with AD-SoS values (*r* = 0.369, *p* = 0.023). No significant associations were observed between handgrip strength and metabolic parameters (*p*_for all_ > 0.05) in patients and controls.

**Figure 1:**
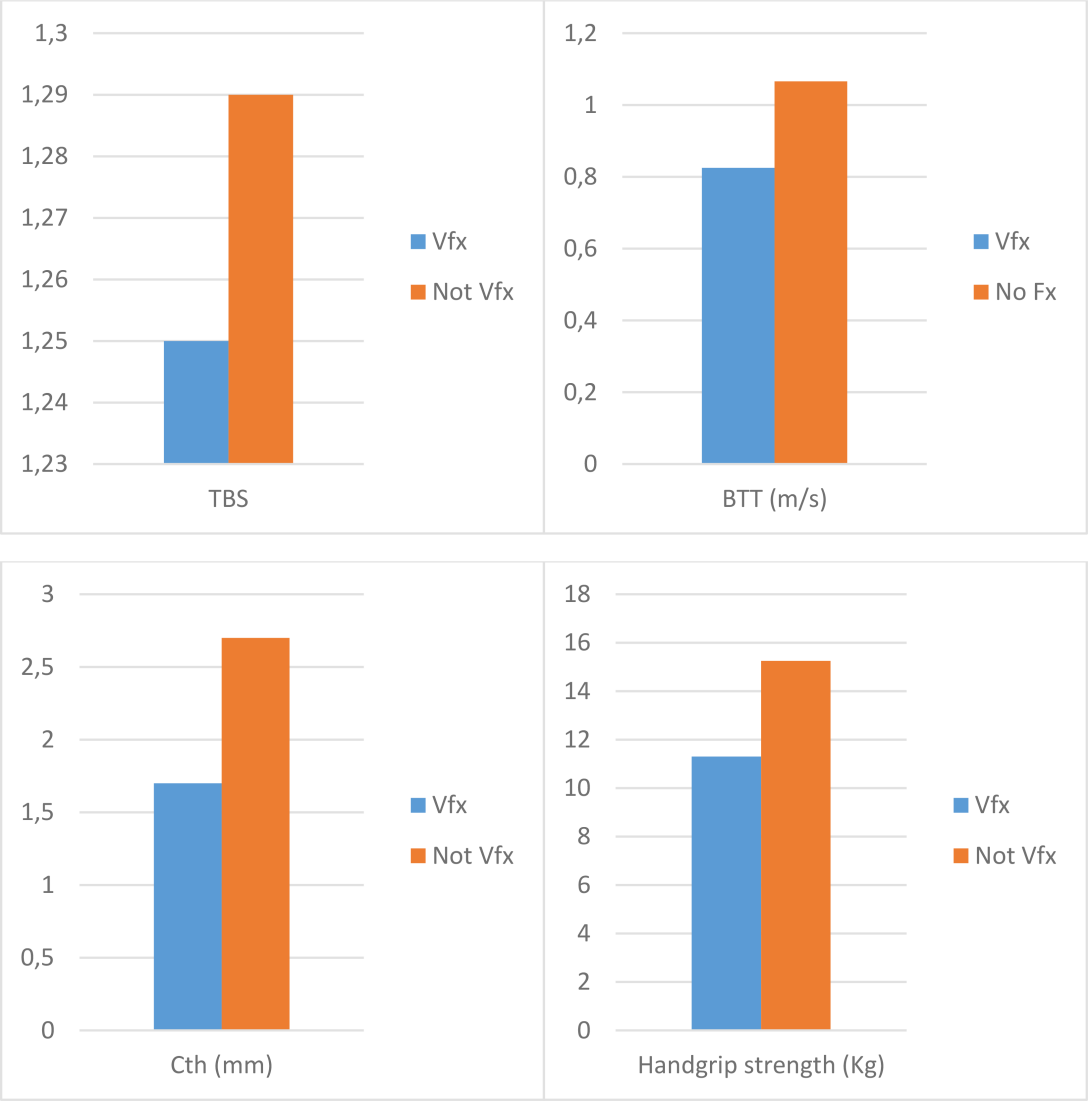
Differences in the trabecular bone score, bone transmission time, cortical thickness, and handgrip strength according to the presence of vertebral fractures in hemodialysis patients. Values are expressed as median and are significantly different between Vfx and not Vfx groups (*p* < 0.05) for all the variables. TBS, trabecular bone score; BTT, bone transmission time; Cth, cortical thickness; Vfx, vertebral fractures.

In the hemodialysis group, 10 participants (24.3%) reported at least one morphometric Vfx; conversely, only six participants (15%) showed Vfx in the control group. In the dialysis group, participants with Vfx compared with participants without Vfx reported significantly different values of TBS, BTT, cortical thickness, and handgrip strength (*p* < 0.05, **Figure 1**).

Finally, we performed a multiple regression analysis, identifying as a dependent variable the 10-year fracture risk for major fracture, whereas age, BMI, time since dialysis, AD-SoS, BTT, cortical bone thickness, TBS, and handgrip strength were included as independent variables. In this analysis, only BTT (*β* = −15.21, SE = 5.91, *p* = 0.02) and TBS (*β* = −54.69, SE = 21.88, *p* = 0.02) turned out to be independently associated with fracture risk. Analysis showed a variance inflation factor for BTT and TBS of 1.624, excluding multicollinearity problems in this model.

## Discussion

This study investigated surrogates of bone strength in hemodialysis patients by the gold-standard DXA and by ultrasonography. Simultaneously, it explored the association between muscle strength and the instrumental surrogates of bone strength, including the DXA-derived TBS. Hemodialysis patients presented poorer bone quality as suggested by reduced TBS and low QUS values compared to controls. Handgrip strength was also lower in hemodialysis patients and was significantly associated with quantitative and qualitative indices of bone strength.

Considering the aging of the CKD population, the relevance of osteoporosis is considerably increasing in this group (21). As a result, adequate screening for bone fragility to prevent the burden of osteoporotic fracture in terms of economic costs, morbidity, and mortality is urgently needed (11). Consequently, the identification of dialysis patients at high fracture risk should be encouraged and included in the diagnostic work-up of CKD patients (31, 32).

Bone fragility encompasses all the characteristics that promote fractures, mainly bone structure, bone turnover, and mineralization deficits that are all observed in CKD (21). However, the association between BMD obtained by DXA, which is the most recognized determinant of bone strength, and fragility fracture observed in CKD patients is not as strong as in the general population (31). Accordingly, Jamal et al. found no association between fractures, hip, and spine BMD; however, tests of neuromuscular function, namely the time up and go, functional reach, and the 6-min walk test, were able to discriminate among patients with and without fractures (25). These observations are consistent with our findings, particularly regarding the association between the handgrip strength and all of the QUS/PEUS surrogates of bone strength. In hemodialysis patients, we observed that participants with reduced handgrip strength were those not only with lower BMD at the lumbar spine and femur but also with lower TBS and AD-SoS, which are accepted markers of bone quality. In particular, TBS and QUS variables have been shown to correlate significantly with bone structural parameters measured by histomorphometry (33).

The reduction in muscle strength could accordingly capture both quantitative and qualitative alterations in bone. The associations shown in **Table 2** between BMD, intended as a quantitative measure of bone strength, and the qualitative indices such as TBS, and also QUS and PEUS, support this hypothesis. These findings also suggest an overall (qualitative/quantitative) bone impairment in hemodialysis patients (34).

Moreover, poorer handgrip strength was noted in the participants with a higher prevalence of morphometric Vfx in the hemodialysis group; conversely, BMD alone was not able to discriminate between hemodialysis patients with Vfx. Consistently, hemodialysis patients, in comparison with the controls, presented BMD values not significantly different at the femoral level and even significantly higher at the lumbar spine. This result highlights that BMD has a low predictive value for fracture in end-stage kidney disease (9).

Since lower muscle performance is also correlated with a higher propensity to fall, it could also explain the higher rate of fractures (35).

Also, due to its simple, inexpensive, rapid, and noninvasive measurement, the handgrip strength evaluation may be considered in the clinical evaluation of CKD to identify patients suitable for further instrumental assessment of bone fragility. For the reason that DXA remains the gold-standard method to check for bone fragility, it appears valuable to also comprise the TBS calculation, at least in dialysis patients (36–40).

In this study, we explored cortical bone by PEUS. Even if no significant differences between dialysis patients and controls were found, consistent with previous data (28, 41), we observed an association between Cth and DI with BMD; DI was also significantly associated with the 10-year probability of femur fracture. The assessment of cortical bone structure in CKD could be critical since it is predominantly affected by the bone loss that is correlated with high PTH and with peripheral fractures (42). BMD measurement by DXA cannot discriminate between cortical and trabecular bone, and PEUS measurement of Cth may contribute, at least in part, to bone status evaluation, even though it is not able to capture microporosity (27).

The 10-year probability of major osteoporotic fractures was independently associated with BTT, suggesting that QUS can help distinct patients at high risk of fracture. QUS measurements have previously been proven to be related to mineral metabolism alterations in hemodialysis (43). Furthermore, the 10-year probability of major osteoporotic fractures was independently inversely associated with TBS, which in turn was strictly related to QUS measurements, probably all of these assessments being dependent on bone quality characteristics such as microarchitecture. Recently, in patients with CKD, Rampersad et al. observed that lower TBS scores were associated with lower eGFR and increased fracture risk in patients with eGFR ≥ 60 ml/min/1.73 m^2^ (44). Nevertheless, the role of TBS in predicting clinical fractures in prospective studies focused on dialysis patients has not yet been demonstrated (45). However, an association with higher bone turnover and prevalent fractures has recently been evidenced in end-stage CKD and dialysis (40, 46, 47).

This research has some limitations represented by the small sample size and the cross-sectional design. On the other hand, it represents the first study to investigate fracture risk by DXA and QUS/PEUS together and to explore the association of muscle strength with indices of bone quality in hemodialysis patients.

In conclusion, we observed a higher fracture risk in hemodialysis patients in comparison with controls. Hemodialysis patients showed lower indices of bone quality, including TBS and phalangeal QUS measurements; moreover, they showed lower handgrip strength. Handgrip strength was associated not only with fracture risk *per se* but also with TBS and phalangeal QUS measurements, which were markers of bone strength and strictly related to the probability of fracture.

Further longitudinal studies looking at the predictive values of surrogates of bone quality on fracture are needed to improve fracture risk management in hemodialysis.

## Data availability statement

The raw data supporting the conclusions of this article will be made available by the authors, without undue reservation.

## Ethics statement

The studies involving human participants were reviewed and approved by the Institutional Research Committee Policlinico G. Martino of Messina. The patients/participants provided their written informed consent to participate in this study.

## Author contributions

AC and NM designed the study. AC, AG, and DS analyzed data and wrote the manuscript. AC, FB, and MF collected data. AX, GG, GB, and GN evaluated the literature included in the study. GS, FC, and AG acquired funding. All the authors had full access to all the data in the study and take responsibility for the integrity and the accuracy of the data analysis. All authors reviewed the manuscript. All authors contributed to the article and approved the submitted version.

## Funding

This study was partially funded by the 2020/2022 Research Plan “Piaceri” of the University of Catania, Open Access line.

## Conflict of interest

The authors declare that the research was conducted in the absence of any commercial or financial relationships that could be construed as a potential conflict of interest.

## Publisher’s note

All claims expressed in this article are solely those of the authors and do not necessarily represent those of their affiliated organizations, or those of the publisher, the editors and the reviewers. Any product that may be evaluated in this article, or claim that may be made by its manufacturer, is not guaranteed or endorsed by the publisher.

## Abbreviations

AD-SoS, amplitude-dependent speed of sound; BMD, bone mineral density; BTT, bone transmission time; CG, Cockcroft–Gault; CKD, chronic kidney disease; CKD-MBD, CKD-mineral and bone disorder; Cth, cortical thickness; CV, coefficient of variation; DI, density index; DXA, dual-energy X-ray absorptiometry; eCcr, estimated creatinine clearance; FRAX, fracture risk assessment tool; FWA, fast-wave amplitude; KDIGO, kidney disease improving global outcomes; NHANES, National Health and Nutrition Examination Survey; PEUS, pulse-echo ultrasonography; QUS, quantitative ultrasonography; SD, standard deviations; SDy, signal dynamic; SoS, speed of sound; TBS, trabecular bone score; UBPI, ultrasound bone profile index; Vfx, vertebral fractures.
